# Gender disparity in maintenance hemodialysis units in South India: a cross-sectional observational study

**DOI:** 10.3389/fneph.2024.1322003

**Published:** 2024-03-25

**Authors:** Mythri Shankar, Gouri Satheesh, Kishan A., Sreedhara C. G., Gireesh G Reddy

**Affiliations:** Department of Nephrology, Institute of NephroUrology, Bengaluru, India

**Keywords:** hemodialysis, gender, disparity, male patient, female patient

## Abstract

**Background:**

Diseases manifest differently according to gender in many medical specialties. However, sex differences in kidney diseases have not been well explored worldwide, especially in India. These differences could also be attributed to sociocultural factors. Although CKD is more prevalent in women worldwide, most men are initiated on kidney replacement therapy (KRT). This study aimed to examine sex disparities in patients on maintenance hemodialysis.

**Materials and methods:**

A cross-sectional observational study was conducted in two maintenance hemodialysis units at the Institute of Nephrourology, a tertiary care referral government center in Bengaluru, India. Demographic characteristics and laboratory parameters were also recorded.

**Results:**

In total, 374 adult patients (aged >18 years) were included in the study. Most patients (72.7%) were men. Mean age in men was 46.95 ± 12.65 years, and women was 46.63 ± 13.66 years. There was no significant difference in marital status and the availability of caretakers between the groups. Spouses were the predominant caretakers for both sexes (64% men and 51% women, P = 0.14). Sons cared more for patients with mother than fathers (19.6% vs 8.8%, P = 0.074). Diabetic nephropathy was the most common cause of ESKD in both groups (33.1% vs 31.3%, P = 0.92). Men had a significantly longer duration of HTN and received more HD sessions per week than women. Mean hemoglobin (9.9 ± 1.79 vs 9.46 ± 1.47 g%) and mean serum creatinine (7.76 ± 2.65 vs 6.41 ± 2.27 mg/dl) were higher in men compared to women (P <0.002). Intradialytic complications, such as hypotension and cramps, were significantly more common in women than in men (P = 0.004). Most men (47.1%) were planning a kidney transplant (and were waitlisted) compared with fewer women (43%). There was no significant difference in the average number of hospitalizations per month or HD vintage.

**Conclusion:**

Women tend to initiate dialysis later, and a lesser number are waitlisted for kidney transplantation, which might be partly related to varying access to or delivery of health care services. Factors such as lack of education, insufficient identification of and strategies to address cultural obstacles to healthcare, and a shortage of financial means to afford medical care are potentially correctable elements that might explain this discrepancy.

## Background

It is well known in many medical specialties that diseases manifest differently according to gender. However, gender differences in kidney diseases have not been well-explored worldwide, especially in India. The differences can be attributed to sociocultural factors. Although CKD is more prevalent in women worldwide, most men are initiated on kidney replacement therapy (KRT), whether dialysis or kidney transplant. Hemodialysis is the major form of KRT worldwide, with 40 women for every 60 men initiated on HD. This study aimed to look at gender disparity in the prevalence and practice of patients on maintenance hemodialysis (1–3).

Similar to many other chronic health conditions, there can be variations in the occurrence of chronic kidney disease (CKD) and end-stage kidney disease (ESKD) as well as differences in how these conditions are treated and the outcomes experienced by men and women. These distinctions may stem from biological dissimilarities between the sexes in normal bodily functions and variances in how healthcare is provided or how aware patients are about chronic kidney disease.

A study conducted within the Dialysis Outcomes and Practice Patterns Study (DOPPS), which represented countries from Europe, the USA, Australia, and New Zealand, indicated that undergoing hemodialysis could eliminate the survival advantage that women typically have over men in the general population. Furthermore, it highlights that fewer women than men receive treatment for end-stage kidney disease (ESKD) despite the higher prevalence of chronic kidney disease in women than in men. Lastly, the use of hemodialysis catheters for vein access is linked to a higher risk of mortality in women than in men (3, 4). There is a lack of data from the Asian population and no data from India regarding the sex disparity in practice patterns of hemodialysis patients. Only one study from Korea showed that women had better survival than men on dialysis due to a lower risk of non-infection and non-cardiovascular related mortality (5). We aimed to evaluate distinctions in hemodialysis-related attributes between sexes in two state government-run hemodialysis centers in South India.

## Methods

This cross-sectional observational study was conducted across two hemodialysis centers in South India: the Institute of Nephro-urology, Bengaluru, India. The data were collected between October 2022 and March 2023. All adult patients (>18 years old) undergoing maintenance hemodialysis for a minimum period of 90 days were included in the study. Sociodemographic characteristics and laboratory parameters were recorded. Patients who died within 90 days of HD initiation and those with incomplete records were excluded from this study. The study was approved by the institutional ethical review board.

## Statistical methods

Descriptive and inferential statistical analyses were performed in the present study. Results on continuous measurements are presented on Mean ± SD (Min–Max) and results on categorical measurements are presented in Number (%). Significance was assessed at the 5% significance level. Student’s t-test (two-tailed, independent) was used to determine the significance of the study parameters on a continuous scale between two groups (intergroup analysis) on metric parameters. Leven’s test for homogeneity of variance has been performed to assess the homogeneity of variance. The t-test was used to compare the means of the two groups. Chi-square and Fisher’s exact tests were used to determine the significance of study parameters on a categorical scale between two or more groups, a non-parametric setting for qualitative data analysis. Fisher’s exact test was used when cell samples were very small.

Significant figures
+Suggestive significance (P-value: 0.05 <P <0.10)
*Moderately significant (P-value:0.01 <P <0.05)
**Strongly significant (P-value: <0.01)

Statistical software: The Statistical software, namely SPSS 22.0, and R environment ver.3.2.2, were used for the analysis of the data and Microsoft Word and Excel have been used to generate graphs, tables, etc. (6–8).

## Results

A total of 374 adult patients (>18 years old) were included in the study. The majority of the patients (72.7%) were men (**Table 1**). Mean age of men was 46.95 ± 12.65 years, and women was 46.63 ± 13.66 years (**Table 2**). The majority were in their fifth decade of life. Almost all the men (99.3%) and women (100%) were married and had caretakers. A total of 94.9% of men and 94.1% of women had caretakers. There were no significant differences in age (P = 0.88) and marital status (P = 1) (**Table 3**). and availability of caretakers (P = 1) between the two groups. Spouses were the predominant caretakers for both sexes (64% for men and 51% for women, P = 0.14). Sons cared more for mothers than fathers (19.6% vs 8.8%, P = 0.074) (**Tables 4**, **5**).

**Table 1:** Gender- frequency distribution of patients studied.

Gender	No. of Patients	%
Male	272	72.7
Female	102	27.3
Total	374	100.0

**Table 2:** Age in Years- frequency distribution of patients studied.

Age in Years	Gender		Total
	Male	Female	
<20	4(1.5%)	0(0%)	4(1.1%)
20-30	26(9.6%)	14(13.7%)	40(10.7%)
31-40	44(16.2%)	18(17.6%)	62(16.6%)
>40	198(72.8%)	70(68.6%)	268(71.7%)
Total	272(100%)	102(100%)	374(100%)
Mean ± SD	46.95±12.65	46.63±13.66	46.86±12.89

P=0.880, Not significant, Student t test.

**Table 3:** Marital status- frequency distribution of patients studied.

Marital status	Gender		Total
	Male	Female	
Married	170(99.3%)	102(100%)	372(99.5%)
Divorced	2(0.7%)	0(0%)	2(0.5%)
Total	172(100%)	102(100%)	374(100%)

P=1.000, Not Significant, Fisher Exact Test.

**Table 4:** Do they have a care taker?- frequency distribution of patients studied.

Do they have a care taker?	Gender		Total
	Male	Female	
No	14(5.1%)	6(5.9%)	20(5.3%)
Yes	258(94.9%)	96(94.1%)	354(94.7%)
Total	272(100%)	102(100%)	374(100%)

P=1.000, Not Significant, Fisher Exact Test.

**Table 5:** If yes, who is the care taker?- frequency distribution of patients studied.

If Yes, who is the care taker?	Gender		Total	P Value
	Male	Female		
SPOUSE	174(64%)	52(51%)	226(60.4%)	0.147
SON	24(8.8%)	20(19.6%)	44(11.8%)	0.074+
MOTHER	18(6.6%)	6(5.9%)	24(6.4%)	1.000
DAUGHTER	16(5.9%)	6(5.9%)	22(5.9%)	1.000
FATHER	18(6.6%)	2(2%)	20(5.3%)	0.290
SELF	14(5.1%)	6(5.9%)	20(5.3%)	1.000
BROTHER	6(2.2%)	4(3.9%)	10(2.7%)	0.614
SISTER	2(0.7%)	2(2%)	4(1.1%)	0.920
DAUGHTER /SON IN LAW	0(0%)	2(2%)	2(0.5%)	0.272
UNCLE	0(0%)	2(2%)	2(0.5%)	0.272
Total	272(100%)	102(100%)	374(100%)	–

Chi-Square Test/Fisher Exact Test.

+Suggestive significance (P-value: 0.05<P<0.10).

Men received more HD sessions per week than women (2.8 ± 0.4 in men vs 2.67 ± 0.59 in women, P-value = 0.075). The average number of yearly physician visits was significantly lower for women (5.44 ± 3.89) than for men (7.34 ± 11.04). Women found it more difficult to buy medications than men (70.6% vs 61%, P = 0.09). Women (53.1%) significantly depended on their spouse for medication expenses compared to men (20.6%) (P = 0.05), while men depended on their income to meet these expenses. Men received higher education than women. The majority (57.2%) had an income of less than INR 20,000 per month, with no significant difference between the groups. Most men (47.1%) planned kidney transplantation surgery compared to women (43%). Women with CKD demonstrated significantly lower awareness regarding their disease state than men with similar conditions (3.2 ± 1.4 versus 21.3 ± 6.2%, respectively; P = 0.004). The average estimated glomerular filtration rate (eGFR) calculated using the CKD EPI formula was 10.6 ml/min/1.73 m^2^ for men and 8.1 ml/min/1.73 m^2^ for women at the time of dialysis initiation. This indicates that women tended to begin dialysis at a more advanced stage of end-stage kidney disease than did men. Only 8% of the patients were initiated on peritoneal dialysis compared to 92% on hemodialysis (P = 0.00). There were no significant sex-specific differences in the dialysis modality. There was no significant difference in the average number of hospitalizations per month and HD vintage between the groups (**Tables 6**–**8**).

**Table 6:** Comparison of clinical variables between men and women.

Variables	Gender		P Value
	Male	Female	
Duration of hypertension (in years)	5.71±5.07	7.25±6.34	0.085+
Frequency of HD (average number of sessions/ week)	2.8±0.4	2.67±0.59	0.075+
HD vintage	1.82±1.99	1.81±1.87	0.969
Average number physician visits per year	7.34±11.04	5.44±3.89	0.093
Average number of hospitalisations in a year	1.84±1.87	1.84±2	0.988

+Suggestive significance (P-value: 0.05<P<0.10).

**Table 7:** Planning for transplantation and access to medications.

PLAN FOR TRANSPLANT	Male	Female	Total	P value
• No	144(52.9%)	58(56.9%)	202(54%)	0.751
• Yes	128(47.1%)	44(43.1%)	172(46%)	
DIFFICULTY IN BUYING MEDICATIONS				
• No	106(39%)	30(29.4%)	136(36.4%)	0.098
• Yes	166(61%)	72(70.6%)	238(63.6%)	
Total	272(100%)	102(100%)	374(100%)	

Chi-Square Test/Fisher Exact Test.

+Suggestive significance (P-value: 0.05<P<0.10); **Strongly significant (P-value: <0.01).

**Table 8:** Socioeconomic parameters.

Variables	Gender		Total	P Value
	Male	Female		
SOURCE OF INCOME				
• SELF	98(36%)	10(9.8%)	108(28.9%)	<0.001**
• SON	54(19.9%)	32(31.4%)	86(23%)	0.140
• SPOUSE	56(20.6%)	44(43.1%)	100(26.7%)	0.05
• FATHER	22(8.1%)	6(5.9%)	28(7.5%)	0.761
• BROTHER	14(5.1%)	6(5.9%)	20(5.3%)	0.084+
• DAUGHTER	12(4.4%)	2(2%)	14(3.7%)	0.675
• MOTHER	6(2.2%)	0(0%)	6(1.6%)	0.563
• SISTER	6(2.2%)	0(0%)	6(1.6%)	0.563
• RELATIVES	4(1.5%)	0(0%)	4(1.1%)	1.000
• GRANDFATHER	0(0%)	2(2%)	2(0.5%)	0.272
EDUCATIONAL STATUS				
• Degree	46(16.9%)	8(7.8%)	54(14.4%)	0.180
• High School	66(24.3%)	32(31.4%)	96(26.2%)	0.423
• Lower Primary	26(9.6%)	4(3.9%)	30(8.1%)	0.365
• Pre university	44(16.2%)	14(13.7%)	58(15.5%)	0.862
• Uneducated	54(19.9%)	32(31.4%)	86(23%)	0.140
• Upper primary	36(13.2%)	12(11.8%)	48(12.8%)	1.000
INCOME PER MONTH				
• <20K	160(58.8%)	54(52.9%)	214(57.2%)	0.463
• >20K	54(19.9%)	16(15.7%)	70(18.7%)	0.463
• Variable/ not fixed	54(19.9%)	28(27.5%)	82(21.9%)	
• Total	272(100%)	102(100%)	374(100%)	

Chi-Square Test/Fisher Exact Test.

+Suggestive significance (P-value: 0.05<P<0.10); **Strongly significant (P-value: <0.01).

Diabetic kidney disease was the most common cause of ESKD in both the men and women (33.1% vs 35.3%, P = 0.92). Congenital anomaly of kidney and urinary tract (CAKUT) and focal segmental glomerulosclerosis (FSGS) as causes for ESKD were particularly common in men, whereas autoimmune diseases such as lupus nephritis were common in women (**Table 9**). Men had a longer duration of hypertension than women (7.25 ± 6.34 years vs 5.71 ± 5.07 years, 0.056). A total of 9.6% of men had ischemic heart disease compared to 2% of women (P = 0.117) (**Table 10**). Mean hemoglobin (9.9 ± 1.79 vs 9.46 ± 1.47 g%) and mean serum creatinine (7.76 ± 2.65 vs 6.41 ± 2.27 mg/dl) were higher in men compared to women (P <0.002). The mean BMI in men was 23.9 ± 3.1 kg/m^2^ and in women, it was 22.8 ± 3.4 kg/m^2^. The mean serum creatinine level was significantly lower in women than in men (6.41 ± 2.27 mg/dl vs 7.76 ± 2.65 mg/dl, P = 0.002). Generally, women tend to have less muscle mass, consume less protein, and experience a greater reduction in body mass, particularly after menopause, than men. Consequently, this may lead to decreased creatinine level (**Table 11**). Intradialytic complications, such as hypotension and cramps, were significantly more common in women than in men (P = 0.004) (**Table 12**). There was no significant difference in the type of vascular access between the groups; however, at the time of hemodialysis initiation, the majority of women were on temporary HD catheters without AV fistula creation (**Table 13**).

**Table 9:** Native Kidney disease- frequency distribution of patients studied.

Native Kidney disease	Gender		Total	P Value
	Male	Female		
Diabetic Kidney Disease	90(33.1%)	36(35.3%)	126(33.7%)	0.920
Chronic Interstitial Nephritis	66(24.3%)	26(25.5%)	92(24.6%)	1.000
Chronic Glomerulosclerosis	46(16.9%)	16(15.7%)	62(16.6%)	1.000
IgA Nephropathy	24(8.8%)	12(11.8%)	34(9.8%)	0.567
Congenital anomaly of kidney and urinary tract (CAKUT)	12(4.4%)	0(0%)	12(3.2%)	0.191
Focal segmental glomerulosclerosis (FSGS)	10(3.7%)	0(0%)	10(2.7%)	0.325
Small and non-biopsible kidneys	18(6.6%)	6(5.9%)	24(6%)	1.000
Autosomal dominant polycystic kidney disease (ADPKD)	4(1.5%)	2(2%)	6(1.6%)	1.000
Chronic pyelonephritis (CPN)	0(0%)	2(2%)	2(0.5%)	0.271
Multiple Myeloma	1(0.7%)	0(0%)	2(0.5%)	1.000
Thrombotic Microangiopathy (TMA)	0(0%)	2(2%)	2(0.5%)	0.272
Total	272(100%)	102(100%)	374(100%)	–

Chi-Square Test/Fisher Exact Test.

**Table 10:** Hypertension and Ischemic heart disease - frequency distribution of patients studied.

HTN	Gender		Total
	Male	Female	
No	6(2.2%)	0(0%)	6(1.6%)
Yes	266(97.8%)	102(100%)	368(98.4%)
Total	272(100%)	102(100%)	374(100%)

P=0.563, Not Significant, Fisher Exact Test.

**Table 11:** Comparison of investigating variables in males and females.

Variables	Gender		Total	P Value
	Male	Female		
HB (g/dl)	9.9±1.79	9.46±1.47	9.78±1.72	0.123
TC (cells/mm3)	6890.21±2405.01	6644.06±2255.03	6823.07±2361.6	0.527
PLATELET (cells/mm3)	2.17±0.66	2.14±0.76	2.16±0.69	0.787
S.CREATININE (mg/dl)	7.76±2.65	6.41±2.27	7.39±2.62	0.002**
UREA (mg/dl)	65.73±28.34	59.08±26.03	63.91±27.82	0.146
Sodium (mEq/L)	138.57±3.36	137.8±4.18	138.36±3.6	0.199
Potasium (mEq/L)	4.71±0.68	4.85±0.71	4.75±0.69	0.226
Chloride (mEq/L)	104.32±4.58	105.18±3.71	104.56±4.37	0.236
Bicarbonate (mEq/L)	19.46±2.95	20.1±2.55	19.63±2.86	0.172
S.Albumin (g/L)	3.88±1.89	3.75±0.44	3.84±1.63	0.649
S.calcium (mg/dl)	8.47±0.83	8.74±0.63	8.55±0.79	0.037*
S.Phosphorous (mg/dl)	4.47±1.08	4.71±1.07	4.53±1.08	0.169
iPTH (ng/L)	621.38±391.52	665.88±474.69	633.81±415.52	0.522
ALKALINE PHOSPHATASE (U/L)	140.65±109.61	152.72±134.33	143.94±116.62	0.530

*Moderately significant (P-value: 0.01<P<0.05); **Strongly significant (P-value: <0.01).

**Table 12:** Complications during hemodialysis.

COMPLICATIONS DURING HD	Gender		Total (n=374)
	Male (n=272)	Female (n=102)	
NO	130(47.8%)	40(39.2%)	170(45.5%)
YES	142(52.2%)	62(60.8%)	204(54.5%)
• CRAMPS	68(25%)	36(35.3%)	104(27.8%)
• INTRA DIALYTIC HYPOTENSION	30(11%)	4(3.9%)	34(9.1%)
• INTRA DIALYTIC HYPERTENSION	8(2.9%)	6(5.9%)	14(3.7%)
• HYPOGLYCEMIA	4(1.5%)	0(0%)	4(1.1%)
• HEADACHE	2(0.7%)	0(0%)	2(0.5%)

P=0.377, Not Significant, Chi-Square Test.

**Table 13:** Vascular access type.

Variables	Gender		Total	P Value
	Male	Female		
VASCULAR ACCESS TYPE				
• AV Fistula	247 (91.1%)	99 (97%)	346 (93%)	1.00
• AV Graft	2(0.7%)	1(1%)	3(0.8%)	1.000
• Tunnelled Catheter	22 (8.1%)	2(2%)	24(6.43%)	0.9

## Discussion

The percentage of women starting hemodialysis in this study was 27.3%, which is grossly disproportionate to men, even though there is a higher prevalence of women with chronic kidney disease (CKD), as evidenced by a systematic review of population-based studies (9) and a recent meta-analysis (10). Potential biological reasons, such as rapid progression in men (11–15), might explain this occurrence. However, there is noteworthy opposing evidence (16, 17). Several analyses revealed a quicker progression in men (11, 12, 14), along with specific studies (18, 19) cited in a crucial meta-analysis (13), which did not solely utilize a decline in glomerular filtration rate but also initiation of KRT to determine ‘progression to ESKD. For women, the delay in starting dialysis and increased mortality rates prior to beginning hemodialysis could be due to delays in seeking medical attention and differences in how decisions regarding starting dialysis are made. In a study that aligned with our findings, Iseki et al. conducted widespread screening using dipstick urinalysis and blood pressure checks. They observed that over a 10-year follow-up period in their Japanese cohort, a smaller proportion of women than men underwent renal replacement therapy (20). Registry data corroborated these disparities, indicating that psychological, social, and economic factors could heavily influence decisions regarding hemodialysis initiation (21). Additionally, women were less knowledgeable about chronic kidney disease and tended to start hemodialysis later than men (22). These observations align with prior research demonstrating sex-specific differences at the start of hemodialysis treatment. Moreover, a recent study analyzing U.S. data by the Arbor Research Collaborative for Health found that women had a slightly lower adjusted estimated glomerular filtration rate (eGFR) than men at the time they began dialysis (23), highlighting a sex disparity in the condition of patients at the commencement of treatment. The prevalence of women initiating dialysis using a catheter more frequently than men might indicate their reduced and delayed access to nephrology care before beginning dialysis, which could in turn contribute to diminished survival rates.

This study indicated that women had fewer physician visits than men. Research undertaken by specialists from India and Harvard University reveal that Indian women encounter gender prejudice when seeking health care. In addition, gender stereotypes inhibit women from expressing their health concerns. The study also determined a direct correlation between travel expenses and women’s access to healthcare. As the distance between a female patient and a hospital increases, the likelihood of women seeking healthcare decreases (24).

Women were less educated than were men in this study. Indian women do not receive education on an equal basis. Even though literacy rates are on the rise, the literacy rate for women, at 65.46%, still falls behind the literacy rate for men at 82.14% (25). One foundational reason, especially in rural areas, for such depressed literacy rates stems from parents’ belief that educating girls is a squandering of resources given that daughters will ultimately reside with their husbands’ families. Consequently, a pervasive sentiment exists that daughters will not directly reap the rewards of educational investments due to their conventional responsibilities and roles as homemakers (26).

This study showed that women depended on the male members of the family (spouse or brother) for medication expenses and faced greater difficulty in procuring the same. The existing participation rate of Indian women in the workforce is 19.2%, whereas that of men is 72%. This reflects a disparity in the gap exceeding 50 percentage points. Women desiring to work face more challenges in securing employment than men, a difficulty that is notably pronounced in Northern Africa and the Arab States, where over 20% of women are unemployed. Although both genders encounter substantial instances of vulnerable employment, women are typically more prevalent in precarious positions. While men are commonly self-employed, women often assist within their family households or relatives’ enterprises (27).

The root causes of chronic kidney disease (CKD) vary between sexes; while diabetes and hypertension tend to be more common in men, autoimmune diseases such as systemic lupus erythematosus are often a more frequent cause of CKD in women, which is consistent with this study (24). Although attributes related to sex contribute to a quicker progression of CKD in men, sex-influenced social and cultural disparities result in a delayed start or absence of kidney replacement therapy in women, including a later referral for kidney transplant evaluation. This study also showed that fewer women were waitlisted for kidney transplantation than were men, which is in agreement with previous studies (23, 28).

In this study, only 19% of the women undergoing dialysis were enrolled in the kidney transplant waitlist, in contrast to 33% of the men. According to the Organ Procurement and Transplantation Network (OPTN), as of 2021, women constituted only 38% of the waitlist (WL). Data from the United States Renal Data System (USRDS) indicated that by 2013, the time spent on WL was 47.7% for men and 49.4% for women. This sex discrepancy persists across various organ waitlists. In Argentina, as of 1 January 2023, only 18% (5,407 individuals) of dialysis patients aged 18 years and above were on the WL, 45.4% (2,456) were women, and 54.6% (2,951) were men (29, 30). Numerous studies indicate that women with End-Stage Kidney Disease (ESKD) have 10% to 20% reduced access to kidney transplants compared to men, even after accounting for demographic and clinical traits (31–33). There are various reasons for women’s underrepresentation in kidney transplant waiting lists. Research reveals that healthcare providers, including physicians, often perceive women as more frail than men, and hence, are less likely to discuss kidney transplantation as a preferred option for kidney replacement therapy with women in advanced stages of CKD or ESKD (34). One study noted that women were 1.45 times more likely not to engage in discussions with healthcare professionals regarding kidney transplantation as a treatment option (35). The disparity was even more pronounced among older women; women aged 66 to 75 years had 29% less access to kidney transplants than men (relative risk, 0.71; 95% confidence interval [CI], 0.68–0.75; P <.001), and women over 75 years had 59% less access than their male counterparts (relative risk, 0.41; 95% CI, 0.34–0.50; P <.001) (32).

The grave implications of these disparities are underscored by a report from the same study showing that women of all ages experienced a similar or even enhanced survival benefit after kidney transplantation compared to men (32).

**Table:** 

DURATION OF HYPERTENSION (YEARS)	Gender		Total (n=184)
	Male (n=133)	Female (n=51)	
<1	22(8.3%)	0(0%)	22(6%)
1-3	98(36.8%)	32(31.4%)	130(35.3%)
>3	152(57.1%)	70(68.6%)	222(60.3%)

P=0.056+, Significant, fisher Exact Test.

**Table:** 

Ischemic heart disease	Gender		Total
	Male	Female	
No	246(90.4%)	100(98%)	346(92.5%)
Yes	26(9.6%)	2(2%)	28(7.5%)
Total	272(100%)	102(100%)	374(100%)

P=0.117, Not Significant, Chi-Square Test.

In this study, a wide majority of the patients were on hemodialysis compared with peritoneal dialysis, with no significant differences in sex. The introduction of the Pradhan Mantri National Dialysis Programme (PMNDP) in 2016 marked a significant step towards ensuring universal access to kidney replacement therapy (KRT) (36). Nonetheless, the challenge of developing a sustainable dialysis delivery model persists due to the limited funding allocated to this initiative. Initially, the emphasis was on establishing HD services; by 2019, PD was included in the scope of public funding. However, funding for HD has been greater than that for PD.

Multiple studies, including the Indian chronic kidney disease study (ICKD), consistently show that women with chronic kidney disease (CKD) experience a lower health-related quality of life (HRQOL) than men. A comprehensive analysis of sex and gender differences in CKD outcomes, including HRQOL, corroborates these observations. Women undergoing maintenance dialysis have reported experiencing a higher number of symptoms and more severe symptoms (4). Furthermore, even after receiving a kidney transplant, the improvement in HRQOL for women was not as significant as that for men. The response to the disease varies significantly between genders, with women being more likely to suffer from depression and anxiety and more inclined to seek emotional and social support to manage their condition. The study suggests that differences in perceived stress and the employment of both maladaptive and adaptive coping mechanisms between men and women could account for some of the disparities in HRQOL (37). Additionally, social and cultural support systems may differ between men and women, with women potentially facing stigma and isolation from the family and society during severe illness or when they are unable to perform traditional caregiving roles, negatively impacting their perceived HRQOL. Women, especially those in rural areas, who may be illiterate, unemployed, and have limited autonomy and decision-making power, face unique challenges that can further diminish their HRQOL when coping with chronic illnesses (38).

Limitations: This study was limited to two hemodialysis centers in South India. Additional research is required to verify whether these findings are true in various countries and with alternative types of kidney replacement therapy.

## Conclusion

In conclusion, it appears that women tend to initiate dialysis later and a lesser number are waitlisted for kidney transplantation, which might be partly related to varying access to or delivery of healthcare services. Factors such as lack of education, insufficient identification of and strategies to address cultural obstacles to healthcare, and a shortage of financial means to afford medical care are potentially correctable elements that might explain this discrepancy.

## Data availability statement

The original contributions presented in the study are included in the article/supplementary material. Further inquiries can be directed to the corresponding author.

## Author contributions

MS: Conceptualization, Methodology, Writing – original draft. GS: Data curation, Writing – review & editing. SC: Resources, Writing – review & editing. KA: Resources, Writing – review & editing. GR:.

## References

[1] ERA-EDTA Registry. ERA-EDTA registry annual report 2015 (ERA-EDTA registry, 2017).

[2] United States Renal Data System. USRDS annual data report: epidemiology of k.

[3] Hecking M Bieber BA Ethier J Kautzky-Willer A Sunder-Plassmann G Säemann MD. Sex-specific differences in hemodialysis prevalence and practices and the male-to-female mortality rate: the Dialysis Outcomes and Practice Patterns Study (DOPPS). PLoS Med. (2014) 11(10):e1001750. doi: 10.1371/journal.pmed.1001750 25350533 PMC4211675

[4] Carrero JJ Hecking M Chesnaye NC Jager KJ. Sex and gender disparities in the epidemiology and outcomes of chronic kidney disease. Nat Rev Nephrol. (2018) 14:151–64. doi: 10.1038/nrneph.2017.181 29355169

[5] Jung HY Jeon Y Kim YS Kang SW Yang CW Kim NH. Sex disparities in mortality among patients with kidney failure receiving dialysis. Sci Rep. (2022) 12:18555. doi: 10.1038/s41598-022-16163-w 36329070 PMC9633833

[6] Suresh KP Chandrasekhar S. Sample Size Estimation and Power analysis for Clinical research studies. J Hum Reprod Sci. (2012) 5:7–13. doi: 10.4103/0974-1208.97779 22870008 PMC3409926

[7] IBM Corp. IBM SPSS Statistics for Windows, Version 22.0. Armonk, NY: IBM Corp (2013).

[8] R Core Team. R: A language and environment for statistical computing. Vienna, Austria: R Foundation for Statistical Computing (2013).

[9] Zhang QL Rothenbacher D. Prevalence of chronic kidney disease in population-based studies: systematic review. BMC Public Health. (2008) 8:117. doi: 10.1186/1471-2458-8-117 18405348 PMC2377260

[10] Hill NR Fatoba ST Oke JL Hirst JA O'Callaghan CA Lasserson DS. Global prevalence of chronic kidney disease—a systematic review and meta-analysis. PLoS One. (2016) 11(7):e0158765. doi: 10.1371/journal.pone.0158765 27383068 PMC4934905

[11] Eriksen BO Ingebretsen OC. The progression of chronic kidney disease: a 10-year population-based study of the effects of gender and age. Kidney Int. (2006) 69:375–82. doi: 10.1038/sj.ki.5000058 16408129

[12] Evans M Fryzek JP Elinder CG Cohen SS McLaughlin JK Nyrén O. The natural history of chronic renal failure: results from an unselected, population-based, inception cohort in Sweden. Am J Kidney Dis. (2005) 46(5):863–70. doi: 10.1053/j.ajkd.2005.07.040 16253726

[13] Neugarten J Acharya A Silbiger SR. Effect of gender on the progression of nondiabetic renal disease: a meta-analysis. J Am Soc Nephrol. (2000) 11:319–29. doi: 10.1681/ASN.V112319 10665939

[14] Pscheidt C Nagel G Zitt E Kramar R Concin H Lhotta K. Sex- and time-dependent patterns in riskfactors of end-stage renal disease: a large Austrian cohort with up to 20 years of follow-up. PLoS One. (2015) 10(8):e0135052. doi: 10.1371/journal.pone.0135052 26322515 PMC4555650

[15] Halbesma N Brantsma AH Bakker SJ Jansen DF Stolk RP De Zeeuw D. Gender differences in predictors of renal function decline in the general population. Kidney Int. (2008) 74(4):505–12. doi: 10.1038/ki.2008.200 18496511

[16] Pani A Bragg-Gresham J Masala M Piras D Atzeni A Pilia MG. Prevalence of CKD and its relationship to eGFR-related genetic loci and clinical risk factors in the Sardinia study cohort. J Am Soc Nephrol. (2014) 25(7):1533–44. doi: 10.1681/ASN.2013060591 PMC4073426 24511125

[17] Jafar TH Schmid CH Stark PC Toto R Remuzzi G Ruggenenti P. The rate of progression of renal disease may not be slower in women compared with men: a patient-level meta-analysis. Nephrol Dial Transplant. (2003) 18(10):2047–53. doi: 10.1093/ndt/gfg317 13679479

[18] Ruggenenti P Gaspari F Perna A Remuzzi. Cross-sectional longitudinal study of spot morning urine protein: creatinine ratio, 24-hour urine protein excretion rate, glomerular filtration rate, and end-stage renal failure in chronic renal disease in patients without diabetes. BMJ. (1998) 316(7310):504–9. doi: 10.1136/bmj.316.7130.504 PMC2665663 9501711

[19] Rosman JB Langer K Brandl M Piers-Becht TP van der Hem GK ter Wee PM. Protein-restricted diets in chronic renal failure: a four-year follow-up shows limited indications. Kidney Int Suppl. (1989) 27:S96–102. 2636680

[20] Iseki K Iseki C Ikemiya Y Fukiyama K. Risk of developing end-stage renal disease in a cohort of mass screening. Kidney Int. (1996) 49:800–5. doi: 10.1038/ki.1996.111 8648923

[21] Faruque LI Hemmelgarn BR Wiebe N Manns BJ Ravani P Klarenbach S. Factors associated with initiation of chronic renal replacement therapy for patients with kidney failure. Clin J Am Soc Nephrol. (2013) 8(8):1327–35. doi: 10.2215/CJN.10721012 PMC3731909 23833317

[22] Coresh J Byrd-Holt D Astor BC Briggs JP Eggers PW Lacher DA. Chronic kidney disease awareness, prevalence, and trends among U.S. adults, 1999 to 2000. J Am Soc Nephrol. (2005) 16(1):180–8. doi: 10.1681/ASN.2004070539 15563563

[23] Kausz AT Obrador GT Arora P Ruthazer R Levey AS Pereira BJG. Late initiation of dialysis among women and ethnic minorities in the United States. J Am Soc Nephrol. (2000) 11(12):2351–7. doi: 10.1681/ASN.V11122351 11095658

[24] Kalra RJ. Access to health care is difficult for most Indian women (2019). Available online at: https://www.dw.com/en/access-to-health-care-a-distant-dream-for-most-Indian-women/a-50108512.

[25] Population Census Data, India. Literacy in India (2022), Census2011.co.in. Retrieved 10 September 2012.

[26] Jamwal Dr. Shubhra Regin Silvest Dr. R. S., Women's Education in India. (2022). Retrieved 10 September 2012. Tamilnadu, India: Island Publishers

[27] International Labour Organization. Available online at: https://www.ilo.org/global/lang–en/index.htm.

[28] Mauvais-Jarvis F Bairey Merz N Barnes PJ Brinton RD Carrero JJ DeMeo DL. Sex and gender: modifiers of health, disease, and medicine. Lancet. (2020) 396:565–82. doi: 10.1016/S0140-6736(20)31561-0 PMC7440877 32828189

[29] Vongsanim S Davenport A. The effect of gender on survival for hemodialysis patients: why don’t women live longer than men? Semin Dial. (2019) 32:438–43. doi: 10.1111/sdi.12817 31044468

[30] Sleiman J Soler Pujol G Montañez E Roatta V Laham G. Access to treatment in chronic kidney disease, dialysis and transplantation. Is there gender equality? Front Med. (2023) 10:1176975. doi: 10.3389/fmed.2023.1176975 PMC10321413 37415763

[31] Bloembergen WE Mauger EA Wolfe RA Port FK. Association of gender and access to cadaveric renal transplantation. Am J Kidney Dis. (1997) 30:733–8. doi: 10.1016/s0272-6386(97)90076-7 9398115

[32] Segev DL Kucirka LM Oberai PC Parekh RS Boulware LE Powe NR. Age and comorbidities are effect modifiers of gender disparities in renal transplantation. J Am Soc Nephrol. (2009) 20:621–8. doi: 10.1681/ASN.2008060591 PMC2653677 19129311

[33] Wolfe RA Ashby VB Milford EL Bloembergen WE Agodoa LY Held PJ. Differences in access to cadaveric renal transplantation in the United States. Am J Kidney Dis. (2000) 36:1025–33. doi: 10.1053/ajkd.2000.19106 11054361

[34] Salter ML Gupta N Massie AB McAdams-DeMarco MA Law AH Jacob RL. Perceived frailty and measured frailty among adults undergoing hemodialysis: a cross-sectional analysis. BMC Geriatr. (2015) 15:52. doi: 10.1186/s12877-015-0051-y 25903561 PMC4428253

[35] Salter ML McAdams-Demarco MA Law A Kamil RJ Meoni LA Jaar BG. Age and sex disparities in discussions about kidney transplantation in adults undergoing dialysis. J Am Geriatr Soc. (2014) 62:843–9. doi: 10.1111/jgs.12801 PMC4024077 24801541

[36] National Health Portal of India. Pradhan Mantri National Dialysis Programme (2018). Available online at: https://www.nhp.gov.in/pradhan-mantri-national-dialysis-programme_pg (Accessed 18 Feb 2024).

[37] Gemmell LA Terhorst L Jhamb M Unruh M Myaskovsky L Kester L. Gender and racial differences in stress, coping, and health-related quality of life in chronic kidney disease. J Pain Symptom Manage. (2016) 52:806–12. doi: 10.1016/j.jpainsymman.2016.05.029 PMC5156935 27697565

[38] Nyamathi AM Ekstrand M Yadav K Ramakrishna P Heylen E Carpenter C. Quality of life among women living with HIV in rural India. J Assoc Nurses AIDS Care. (2017) 28:575–86. doi: 10.1016/j.jana.2017.03.004 PMC5468467 28473182

